# The Effect of Fermented Lingonberry Juice on* Candida glabrata* Intracellular Protein Expression

**DOI:** 10.1155/2017/6185395

**Published:** 2017-03-30

**Authors:** Pirjo Pärnänen, Ali Nawaz, Timo Sorsa, Jukka Meurman, Pirjo Nikula-Ijäs

**Affiliations:** ^1^Department of Oral and Maxillofacial Diseases, Helsinki University Hospital (HUH), University of Helsinki, Haartmaninkatu 8, Box 63, 00014 Helsinki, Finland; ^2^Department of Biosciences, Division of Biochemistry and Biotechnology, University of Helsinki, Viikinkaari 5D, 00790 Helsinki, Finland; ^3^Division of Periodontology, Department of Dental Medicine, Karolinska Institutet, Box 4064, 141 04 Huddinge, Sweden

## Abstract

Lingonberries have a long traditional use in treating fungal infections on mucosal membranes, but very little is known about the exact antifungal mechanisms. We tested the effects of fermented lingonberry juice on* Candida glabrata* intracellular protein expression. A* Candida glabrata* clinical strain was grown in the presence of fermented lingonberry juice (FLJ). Also the effect of lowered pH was tested. Intracellular protein expression levels were analyzed by the 2D-DIGE method. Six proteins detected with ≥1.5-fold lowered expression levels from FLJ treated cells were further characterized with LC-MS/MS. Heat shock protein 9/12 and redoxin were identified with peptide coverage/scores of 68/129 and 21/26, respectively. Heat shock protein 9/12 had an oxidized methionine at position 56. We found no differences in protein expression levels at pH 3.5 compared to pH 7.6. These results demonstrate that FLJ exerts an intracellular stress response in* Candida glabrata*, plausibly impairing its ability to express proteins related to oxidative stress or maintaining cell wall integrity.

## 1. Introduction

Oral yeast infections are most commonly caused by* Candida albicans*. Predisposing factors include the use of broad-spectrum antibiotics, dry mouth, or ill-fitting dental prostheses. The second most common yeast* Candida glabrata* (*C. glabrata*) is an opportunistic fungal pathogen and causes serious infections, particularly in the immunocompromised patients [1, 2].* C. glabrata's *resistance to the most commonly used antifungals, the azoles, is considered to be innate or acquired [3]. Additionally,* C. glabrata* biofilms are more resistant to antifungals [4].* C. glabrata* has been known to damage the host's immune responses by inducing proinflammatory cytokines [5] as well as modulating proteolysis [6, 7]. The search for new antifungal agents leads us to test the effects of fermented lingonberry juice on* C. glabrata*.

There are a few studies on the effects of lingonberries on* C. albicans* and other oral microbial species, but the effects on* C. glabrata* have not been tested. Most of these studies concern antimicrobial, biofilm formation, or adhesion/coaggregation properties [8–10]. The effects on intracellular protein expression by* C. glabrata* have not been addressed with lingonberry. Lingonberries are rich in phenolic compounds, which are thought to be beneficial to health. The antimicrobial fractions from lingonberries have been partly solved, but the chemical complexity of the berry material makes it difficult to precisely pinpoint the active ingredient. The aim of our study was to evaluate the effect of fermented lingonberry juice (FLJ) on* C. glabrata *intracellular protein expression.

## 2. Materials and Methods

### 2.1. Yeast Growing and Fermented Lingonberry Juice Treatment

A clinical* C. glabrata* (T-1639) from a patient from Helsinki University Central Hospital was cultured on a Sabouraud dextrose agar plate (SDA plate, Lab M, Bury, UK) for 18 h at 37°C. Two separate colonies were cultured in YPG (0.5% yeast extract, 1% peptone, and 0.5% glucose) o/n at 37°C. The amount of yeast cells was adjusted to 0.6 × 10^7^ CFU/mL. Fermented and lyophilized lingonberry juice was prepared as described by Pärnänen [11]. Three sets of cultures were made: 9 mL YPG pH 7.6 + 1 mL of yeast suspension, 9 mL of YPG pH 3.5 + 1 mL of yeast suspension, and 9 mL YPG pH 7.6 + 1 mL of yeast suspension + 1.05 g freeze-dried FLJ (final pH 3.5). To retrieve enough yeast cells for further protein assays, we found that 1.05 g/10 mL of lingonberry powder and 2.5 h treatment time were appropriate to inhibit 50% of growth. After 2.5 h incubation at 37°C, the yeast cells were washed two times with 10 mL MQ (4000*g*, 10 min, RT). To see if the treatments had a long-term effect, the remaining pellets were suspended into 9 mL of YPG, pH 7.6, and cultured on SDA plates for 18 h at 37°C (vertical shaker, slow speed). After the incubation, the cells were washed two times with 10 mL MQ. The cell pellets were suspended in 1x lysis buffer (8 M urea; 4% CHAPS; 30 mM Tris, pH 8.0) and broken with 0.5 mm glass beads 18 times for 30 s with 30 s intervals (10 min pause on ice after every 6 cycles). The suspension was filtered with a 0.22 *μ*m filter (Millipore).

### 2.2. 2D-DIGE

The suspensions were purified using 2D Clean-Up Kit (GE Healthcare, Uppsala, Sweden) and quantified with 2D Quant Kit (GE Healthcare, Uppsala, Sweden) according to manufacturer's recommendations. The samples were labelled with CyDye DIGE Fluors (minimal dyes) Cy2, Cy3, and Cy5 (GE Healthcare, Buckinghamshire, UK) according to manufacturer's instructions (Table 1). Isoelectric focusing was performed (IPGphor, GE Healthcare) with Immobiline DryStrip, pH 4–7 (GE Healthcare), with values of 150 V 3 h, 300 V 3 h, 1000 V 6 h, 8000 V 1 h 15 min, and 8000 V 3 h 45 min. 2D electrophoresis was performed with values of 150 V, 30 mA, 2 W for 1 h and 500 V, 500 mA, 45 W for 4 h. Three gels were run/colony (total six gels). The gels were scanned with Typhoon 4044, and analyses of the expression level changes of proteins were made with DeCyder 2D Differential Analysis Software (GE Healthcare). All gels were silver-stained after laser scanning. Protein spots exhibiting ≥ 1.5-fold alteration in expression were cut out from the silver-stained gels and stored at −20°C.

### 2.3. LC-MS/MS

Silver-stained protein bands were “in-gel” digested. Cysteine bonds were reduced with 0.045 M dithiothreitol (#D0632, Sigma-Aldrich, USA) for 20 min at 37°C and alkylated with 0.1 M iodoacetamide (#57670 Fluka, Sigma-Aldrich, USA) at room temperature. Samples were digested by adding 0.75 *μ*g trypsin (Sequencing Grade Modified Trypsin, V5111, Promega). After digestion, peptides were purified with C18 microspin columns (Harvard Apparatus) according to manufacturer's protocol and redissolved in 30 *μ*L.

Liquid chromatography coupled to tandem mass spectrometry (LC-MS/MS) analysis was carried out on an EASY-nLC (Thermo Fisher Scientific, Germany) connected to a Velos Pro Orbitrap Elite hybrid mass spectrometer (Thermo Fisher Scientific, Germany) with nanoelectrospray ion source (Thermo Fisher Scientific, Germany). The LC-MS/MS samples were separated using a two-column setup consisting of a 2 cm C18-A1 trap column (Thermo Fisher Scientific, Germany), followed by a 10 cm C18-A2 analytical column (Thermo Fisher Scientific, Germany). The linear separation gradient consisted of 5% buffer B in 5 min, 35% buffer B in 60 min, 80% buffer B in 5 min, and 100% buffer B in 10 min at a flow rate of 0,3 *μ*L/min (buffer A: 0,1% TFA in 1% acetonitrile; buffer B: 0,1% TFA acid in 98% acetonitrile). 6 *μ*L of sample was injected per LC-MS/MS run and analyzed. Full MS scan was acquired with a resolution of 60 000 at normal mass range in the orbitrap analyzer. The method was set to fragment the 20 most intense precursor ions with CID (energy 35). Data was acquired using LTQ Tune software.

Acquired MS2 scans were searched against UniProt* Candida glabrata* protein database using the SEQUEST search algorithms in Thermo Proteome Discoverer. Allowed error for the precursor ions was 15 ppm and mass error for the fragment was 0.8 Da. A static residue modification parameter was set for carbamidomethyl +57,021 Da (C) of cysteine residue. Methionine oxidation was set as dynamic modification +15,995 Da (M). Only full-tryptic peptides were allowed for scoring and maximum of 1 missed cleavage was considered.

## 3. Results

**Figure 1:**
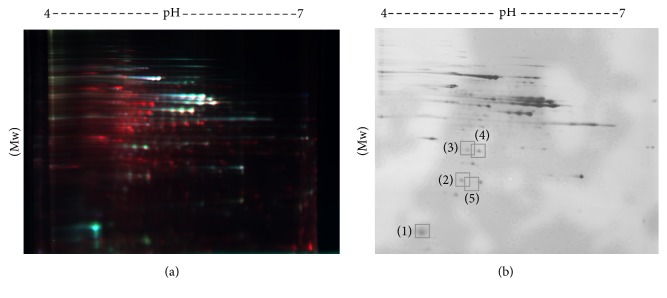
(a) 2D-DIGE gel. Protein spots labelled Cy2 (yellow std), Cy3 (red), and Cy5 (blue) from gel nro 3. Isoelectric focusing (pH 4–7, linear) is shown on top of the figure; MW is shown on the left vertical axis. (b) Silver-stained 2D-DIGE gel. Protein spots cut out of the gel are enumerated and encircled. Corresponding spots are found from (a).

**Figure 2:**
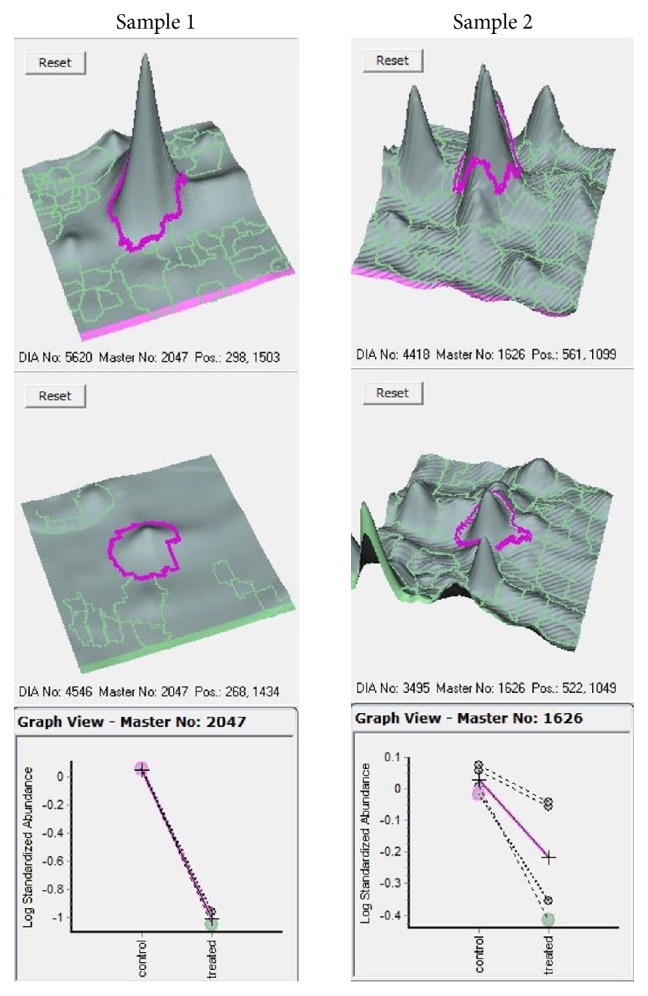
Results from the analysis with DeCyder 2D Differential Analysis Software (GE Healthcare). Sample 1 is HSP 9/12 (Q6FPF6); sample 2 is redoxin (Q6FIU4).

2D-DIGE gel is shown in Figure 1(a). The silver-stained gel is shown in Figure 1(b). The results from the two separate* C. glabrata* T-1639 colonies were similar. There were no significant effects of pH on the intracellular protein expression levels at pH 3.5 compared to pH 7.6 (gels 2 and 5), and because of this we analyzed the rest of the gels as quadruplicate repetitions with Student's *t*-test and one-way ANOVA and achieved significant differences in six proteins. After FLJ treatment, a great number of the hundreds of the intracellular proteins showed elevated or decreased expression levels compared to the control, but mostly the alterations were nonsignificant. Six proteins showed ≥1.5-fold decreased significant expressions. Results from the UniProt protein database search are shown in Table 2. The peptide coverage (%)/scores in one sample were so low (sample 6) that we could not identify the protein. In sample 5, the coverage/score was 12.5/7.7 compared to* C. glabrata* CBS138 glyceraldehyde-3-phosphate dehydrogenase-2 (GADPH-2). Samples 3 and 4 showed coverage/scores of 13.1/11.9 and 13.8/13.1 subsequently for* C. glabrata* CBS138 adenylate kinase. Sample 2 gave coverage/score of 20.6/25.6 and was matched with redoxin Q6FIU4 (Figure 2.). Sample 1 showed the highest values of 68/129 and has methionine oxidation at position 56 and it matches* C. glabrata *CBS138 heat shock protein 9/12 (HSP 9/12) (Q6FPF6) (Figure 2.).

## 4. Discussion

The aim of this study was to find new means to prevent candidosis, especially* C. glabrata*- related infections, by identifying potential candidate proteins which are downregulated due to the treatment with FLJ and to address their roles in* C. glabrata* cell viability and upregulation of oral biofilm formation and thickening subsequently leading to candidosis and related inflammation. Among the studied proteins, five were significantly downregulated and identified with LC-MS/MS. Thus downregulation may eventually cause reduction in their pathological potential to induce disease. The results from our study demonstrate that FLJ exerts anticandidal effects that might have clinical implications.

The inhibition of the* C. glabrata *growth by FLJ was pronounced, and the amount of FLJ as well as treatment time needed to be minimized to obtain enough protein for the analyses. In our study, there was no significant difference in intracellular protein expression at pH 3.5 compared to pH 7.6. Indeed, Ullah et al. [12] have shown that* C. glabrata *is capable of maintaining more stable intracellular pH compared to* S. cerevisiae *when challenged with low extracellular pH. Interestingly, FLJ treatment did have a long-term effect on* C. glabrata *intracellular protein expression.

In fact, here we demonstrate FLJ-dependent downregulation of GADPH-2 in* C. glabrata*. GADPH is involved in the carbohydrate processing and one of the early steps in glycolysis. In* S. cerevisiae*, GADPH activity is associated with exponentially growing cells [13]. Azole resistance associated with petite mutations in* C. glabrata *[14] revealed alterations of intracellular proteins, for example, GADPH-2 and HSP 12.

Adenylate kinase catalyzes the formation of ADP [15]. FLJ caused downregulation of* C. glabrata *adenylate kinase expression, implicating that the ADP availability to be utilized in the oxidative phosphorylation can be diminished, and this may also impair the energy requirements essential for the pathological process leading to candidosis.

In our study, the expressions of HSP 9/12 and redoxin were lowered to the largest degree. HSP 9 from* Saccharomyces pombe* is related to stress response and HSP 12 from* Saccharomyces cerevisiae *maintains cell stability under stress conditions [16]. Redoxin is associated with reduced oxidative stress [17]. In our study, the methionine oxidation of HSP 9/12 after the FLJ- treatment also implies that some oxidation-promoting events on the cell wall have occurred. It has been shown that increased antioxidant proteins of* C. albicans*, for example, alkyl hydroperoxide reductase, thioredoxin peroxidase, and thioredoxin [18], and upregulated stress response proteins of* C. glabrata*, for example, HSP 12 and cytoplasmic thioredoxin isoenzyme (Trx1p), are associated with enhanced biofilm formation compared to planktonic cells.

## 5. Conclusions

Biofilm formation, that is, accumulation of dental plaque, is closely related to upregulated glycolysis or microbial carbohydrate utilization, leading, if uncontrolled or not managed, to the triggered pathological proinflammatory host response. In this regard, oral hygiene and early interventions by removal of accumulating biofilm are essential. Eventually, the current findings indicate that FLJ exerts potential to reduce or even prevent the energy supplies involved in the pathological and excessive biofilm accumulation. This implicates reduced propathogenic potential of biofilm exposed to FLJ.

Bioactive berry compounds are known to cause destabilization of cell membranes of human pathogens [7], causing various antimicrobial effects, for example, depletion of the intracellular ATP pool, inhibition of oxidative phosphorylation, or inhibition of cell wall enzyme activity. Our results demonstrate multiple plausible mechanisms for potential inhibitory effects of fermented FLJ on* C. glabrata* growth related to changes in the intracellular proteome. Further studies are warranted on the mechanisms of interactions on the cell wall and intracellular level as well as on the inhibition of biofilm growth.

## Figures and Tables

**Table 1:** Labelling of the samples with cyanidin dye fluorescent labels (NHS esters): Cy3 and Cy5 for 2D-DIGE. The gels were run in duplicate. Cy2 was used as the internal standard. Cells were treated with fermented lingonberry juice (FLJ).

Gels 1 & 4	pH 3.5	Cy3
	FLJ	Cy5

Gels 2 & 5	pH 3.5	Cy5
	pH 7.6	Cy3

Gels 3 & 6	pH 7.6	Cy3
	FLJ	Cy5

**Table 2:** UniProt *Candida glabrata* protein database search results.

	Accession	Description	Score	Coverage	# proteins	# unique peptides	# peptides	# PSMs	# AAs	MW [kDa]	Calc. pI
Sample 1	Q6FPF6	Strain CBS138 chromosome J complete sequence OS = *Candida glabrata* (strain ATCC 2001/CBS 138/JCM 3761/NBRC 0622/NRRL Y-65) GN = CAGL0J04202 g PE = 4 SV = 1−[Q6FPF6_CANGA]	129,01	67,96	1	8	8	43	103	11,2	5,02
		A2	Sequence	# PSMs	# proteins	# protein groups	Protein group accessions	Modifications	ΔCn	XCorr	Probability
		High	GADEANAESYADTAR	11	1	1	Q6FPF6		0,0000	4,85	0,00
		High	GVAQGMHDSAQK	5	1	1	Q6FPF6		0,0000	3,85	0,00
		High	GVAQGMHDSAQK	10	1	1	Q6FPF6	M6 (oxidation)	0,0000	3,10	0,00
		High	LNEGLTPDSQK	5	1	1	Q6FPF6		0,0000	3,01	0,00
		High	LNDAVEYVSK	5	1	1	Q6FPF6		0,0000	2,99	0,00
		High	FQGEENKGVAQGMHDSAQK	1	1	1	Q6FPF6	M13 (oxidation)	0,0000	2,81	0,00
		High	GKEFVTDETDK	2	1	1	Q6FPF6		0,0000	2,51	0,00
		High	EFVTDETDKLAGK	1	1	1	Q6FPF6		0,0000	2,45	0,00
		Medium	FQGEENK	3	1	1	Q6FPF6		0,0000	2,33	0,00

Sample 2	Q6FIU4	Strain CBS138 chromosome M complete sequence OS = *Candida glabrata* (strain ATCC 2001/CBS 138/JCM 3761/NBRC 0622/NRRL Y-65) GN = CAGL0M11704 g PE = 4 SV = 1−[Q6FIU4_CANGA]	25,57	20,57	1	4	4	9	175	18,9	5,53
		A2	Sequence	# PSMs	# proteins	# protein groups	Protein group accessions	Modifications	ΔCn	XCorr	Probability
		High	VGEGVYWSGR	2	1	1	Q6FIU4		0,0000	3,45	0,00
		High	FATDAGAELVR	5	1	1	Q6FIU4		0,0000	3,33	0,00
		High	HLGYELK	1	1	1	Q6FIU4		0,0000	2,55	0,00
		High	NLGVQNTK	1	1	1	Q6FIU4		0,0000	2,34	0,00
